# Cell-Free Synthesis: Expediting Biomanufacturing of Chemical and Biological Molecules

**DOI:** 10.3390/molecules29081878

**Published:** 2024-04-20

**Authors:** So-Jeong Lee, Dong-Myung Kim

**Affiliations:** Department of Chemical Engineering and Applied Chemistry, Chungnam National University, 99 Daehak-Ro, Daejeon 34134, Republic of Korea; sjlee8807@naver.com

**Keywords:** biomanufacturing, cell-free synthetic biology, metabolic engineering, pathway prototyping, on-demand production

## Abstract

The increasing demand for sustainable alternatives underscores the critical need for a shift away from traditional hydrocarbon-dependent processes. In this landscape, biomanufacturing emerges as a compelling solution, offering a pathway to produce essential chemical materials with significantly reduced environmental impacts. By utilizing engineered microorganisms and biomass as raw materials, biomanufacturing seeks to achieve a carbon-neutral footprint, effectively counteracting the carbon dioxide emissions associated with fossil fuel use. The efficiency and specificity of biocatalysts further contribute to lowering energy consumption and enhancing the sustainability of the production process. Within this context, cell-free synthesis emerges as a promising approach to accelerate the shift towards biomanufacturing. Operating with cellular machinery in a controlled environment, cell-free synthesis offers multiple advantages: it enables the rapid evaluation of biosynthetic pathways and optimization of the conditions for the synthesis of specific chemicals. It also holds potential as an on-demand platform for the production of personalized and specialized products. This review explores recent progress in cell-free synthesis, highlighting its potential to expedite the transformation of chemical processes into more sustainable biomanufacturing practices. We discuss how cell-free techniques not only accelerate the development of new bioproducts but also broaden the horizons for sustainable chemical production. Additionally, we address the challenges of scaling these technologies for commercial use and ensuring their affordability, which are critical for cell-free systems to meet the future demands of industries and fully realize their potential.

## 1. Introduction

The production of chemical products is crucial for modern society, but it also brings significant environmental issues. Traditional chemical production methods depend heavily on finite, non-renewable resources, causing pollution and greenhouse gas emissions that contribute to global climate change. Against this backdrop, there is an urgent demand for sustainable manufacturing practices that can significantly reduce or eliminate the environmental impact of chemical production. Biomanufacturing offers a promising direction by using microorganisms or enzymes and leveraging their efficient catalytic abilities. This method not only improves the specificity and efficiency of chemical production but also uses renewable resources, thus markedly reducing the carbon footprint of the industry.

Efforts to transition to bio-based manufacturing are growing globally, with initiatives like the United States’ BioPreferred Program [1], the European Union’s Biobased Industries Consortium (BIC) [2], Brazil’s RenovaBio program [3], and China’s National Bioindustry Development Plan [4] exemplifying the concerted push towards integrating bio-based sectors and facilitating the market entry of bio-based products.

However, engineering microorganisms for chemical production can be challenging, often requiring a trial-and-error process. Advances in synthetic biology are making this process more efficient, facilitating the creation of microorganisms tailored for high-yield chemical production using standardized parts and automated techniques [5,6,7]. The success of companies such as LanzaTech in bioprocess-based chemical production underscores synthetic biology’s vital role in promoting the bio-based economy [8,9]. Yet, living cells’ complexity and unpredictability pose challenges for process control and consistent production yields. The Design–Build–Test–Learn (DBTL) cycle, essential to synthetic biology’s cell engineering approach, faces hurdles, especially in the transformation and cultivation of cells [10,11,12,13].

Cell-free synthesis systems emerge as a promising solution in this scenario [14,15,16,17]. They enable the direct expression of genes from linear DNA without living cells, offering a flexible and controllable platform for swiftly developing enzymatic pathways. This approach sidesteps challenges like cellular toxicity and membrane barriers, simplifying the testing and optimization of chemical production. Moreover, cell-free synthesis supports the trend towards decentralized production, addressing issues like supply chain vulnerabilities, high energy consumption, and significant environmental impacts associated with centralized manufacturing [18,19,20,21]. Decentralized production, located nearer to raw materials and consumers, promotes sustainability, efficiency, and adaptability. It highlights the benefits of localized, on-site production systems in meeting unique demands and reducing the chemical industry’s environmental footprint.

## 2. Development and Evolution of Cell-Free Synthesis Systems

Cell-free synthesis systems offer a significant advantage over traditional cell-based methods by utilizing isolated cellular machinery. This approach circumvents the complexities and limitations of working with live organisms, facilitating direct programming and flexible configuration of reactions [22]. Initially developed for decoding genetic information, cell-free techniques have roots in the foundational work of Nirenberg and colleagues. Their use of synthetic RNA sequences, like poly U, in crude cell extracts for homopolymeric protein synthesis helped decode the relationship between nucleic acid sequences and amino acid sequences in proteins [23]. This not only illuminated fundamental biological processes but also demonstrated that protein synthesis could be emulated outside of living cells, setting the stage for the production of recombinant proteins in vitro. While initial cell-free systems for codon identification produced only small, analytically detectable amounts of proteins, advancing these systems for recombinant protein production necessitated significant engineering to enhance protein yield and longevity. The pivotal advancements in addressing translational machinery longevity include the development of continuous-flow cell-free (CFCF) and continuous-exchange cell-free (CECF) synthesis systems [24,25]. These innovations ensured a constant supply of substrates and efficient removal of by-products, with subsequent research highlighting that sustained protein production in these setups was supported by a continuous supply of ATP and the removal of inorganic phosphate. These breakthroughs paved the way for the development of high-yield batch cell-free synthesis systems, achieving production yields comparable to continuous configurations by maintaining ATP supply without phosphate accumulation [26]. Employing crude cell extracts in cell-free systems introduces a mix of cellular enzymes that retain their biological activity. Despite seeming less ideal than purified systems, the presence of these enzymes can boost cell-free synthesis efficiency. For example, replicating central carbon metabolism in cell-free systems meets the ATP demand without phosphate buildup. Specifically, in cell-free systems using *E. coli* extracts, glucose metabolism through pathways like glycolysis, the TCA cycle, and the electron transport chain generates more ATP than methods relying on traditional high-energy phosphate sources, such as creatine phosphate, acetylphosphate, and phosphoenolpyruvate [27]. This demonstrates that cell-free synthesis, while distancing from living cell use, can still harness specific cellular functions to enhance system performance. It also indicates that evolving cell-free synthesis strategically involves activating or suppressing pathways present in the cell extract.

**Figure 1:**
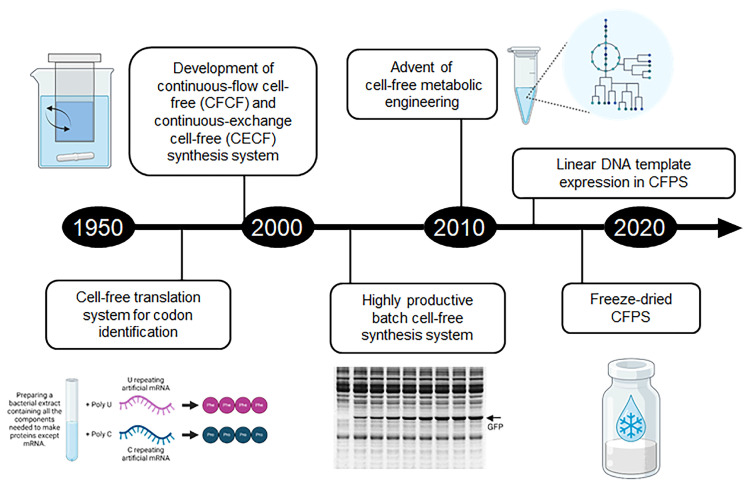
Development and evolution of cell-free synthesis technologies. This figure illustrates the progressive milestones in the field of cell-free synthesis, tracing its journey from an initial analytical tool used to decode codons to its current state, where it can produce milligram quantities of proteins per milliliter of reaction mixture. The advancements highlighted include the improvement of cell-free protein synthesis systems in terms of productivity and cost-effectiveness, the innovative use of linear DNA templates for protein synthesis, and the expansion of cell-free synthesis applications into metabolic engineering. These advancements have facilitated the creation of programmable cell-free metabolic systems. Additionally, the development of lyophilized cell-free systems marks a significant step towards the decentralized and on-demand production of proteins and chemical compounds, opening new avenues for applying cell-free technology in various fields [23,24,25,26].

**Figure 2:**
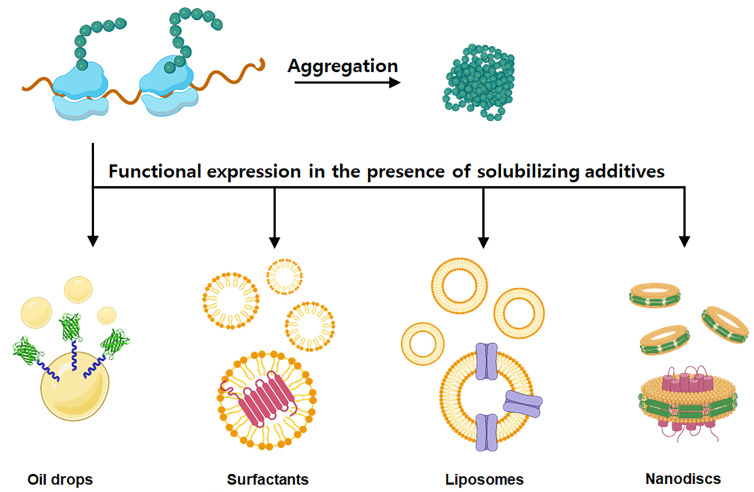
Strategies for membrane protein synthesis in cell-free synthesis systems. The folding and stability of membrane proteins can be improved by adding surfactants or detergents to a cell-free synthesis system to form micelles or directly adding artificial synthetic membranes such as liposomes or nanodiscs to express them. Oils can also be added to anchor the hydrophobic domains of the proteins.

Thanks to these advancements over the last decade (outline in Figure 1), cell-free synthesis systems have evolved from being analytical tools for biological study to versatile platforms capable of generating protein molecules for practical applications. In particular, it provides many advantages for expressing proteins that are difficult to produce using traditional methods, such as membrane proteins. Traditional cell-based approaches often encounter challenges such as low expression levels, protein misfolding, and toxicity to host cells, particularly for complex membrane proteins. Additionally, the manipulation of cellular systems to produce membrane proteins may require labor-intensive optimization steps and specialized equipment, further complicating the process. Moreover, the isolation and purification of membrane proteins from cellular membranes can be challenging and may result in low yields and compromised protein quality. In contrast, cell-free synthetic systems offer several advantages for membrane protein production that address these limitations. Firstly, cell-free systems bypass the need for intact living cells, eliminating the toxicity and metabolic burden associated with traditional cell-based methods. This enables the production of membrane proteins that may be difficult to express or toxic to host cells in a controlled and customizable environment. Secondly, cell-free systems allow for the direct incorporation of membrane components, such as lipids and detergents, facilitating the stabilization and functional reconstitution of membrane proteins in their native environment. This is particularly advantageous for the production of complex membrane proteins that require specific lipid environments for proper folding and stability (Figure 2). Peter et al. applied a simple method of adding oil to a cell-free synthesis system. The membrane protein ssMP can be localized to the surface of the oil to prevent aggregation, and fluorescence could be observed on the oil surface by expressing a protein fused to ssMP and GFP [28]. Justin et al., by introducing a synthetic membrane into a cell-free synthesis system, synthesized the nitrate-sensing membrane protein NarX-L and modified the sensing domain of NarX to create a biosensor that expresses the reporter protein NanoLuc through signaling in the presence or absence of nitrate [29]. Miranda et al. applied diblock copolymers using a new type of synthetic membrane mimetic material rather than natural membrane vesicles and attempted to increase membrane protein production by investigating its effect on protein folding [30].

## 3. Classification of Cell-Free Synthesis Systems

Cell-free systems are classified based on the source of cellular extract and the method of preparation. Prokaryotic cell-free systems utilize extracts from bacteria like *E. coli*, offering simplicity and the high-yield production of proteins. Conversely, eukaryotic cell-free systems employ extracts from organisms such as yeast or mammalian cells, enabling the proper folding of complex proteins. These systems are also potentially suitable for applications requiring eukaryotic-specific modifications. Additionally, cell-free systems can be categorized as crude extract systems, utilizing direct cell lysates, or reconstituted systems, assembled from purified components. Crude extract systems retain the native cellular environment and are cost-effective, while reconstituted systems offer greater control over reaction conditions and enable the study of specific biochemical processes.

## 4. Deploying Synthetic Pathways into Cell-Free Synthesis Systems

**Figure 3:**
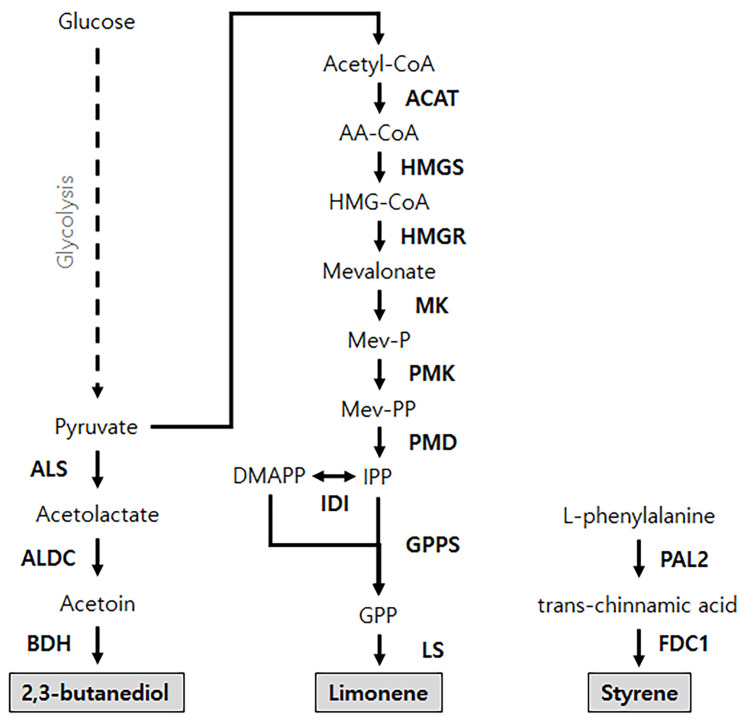
Utilization of cell-free synthesis systems for prototyping metabolic pathways. This figure showcases several examples in which cell-free synthesized enzymes are harnessed to evaluate and optimize designed metabolic pathways aimed at producing valuable chemical compounds. Highlighted are specific case studies where cell-free systems were used to prototype pathways for the synthesis of 2,3-butanediol, limonene, and styrene, illustrating the versatility and efficacy of cell-free synthesis in facilitating the rapid testing and development of novel biochemical pathways for the production of industrially relevant compounds. ALS, acetolactate synthase; ALDC, acetolactate decarboxylase; BDH, 2,3-butanediol dehydrogenase; ACAT, acetyl-CoA acetyltransferase; HMGS, hydroxymethylglutaryl-CoA synthase; HMGR, hydroxymethylglutaryl-CoA reductase; MK, mevalonate kinase; PMK, phosphomevalonate kinase; PMD, pyrophosphomevalonate decarboxylase; IDI, isopentenyl pyrophosphate isomerase; GPPS, geranyl diphosphate synthase; LS, limonene synthase; PAL2, phenylalanine ammonia lyase; FDC1, ferulic acid decarboxylase [34,35,36].

As cell-free protein synthesis technology progresses in both productivity and cost-efficiency, its applications extend well beyond the simple translation of genetic information. The presence of cellular enzymes within cell extracts effectively converts the reaction mixture of a cell-free system into an “open cell,” readily programmable with genes encoding enzymes for specific pathways. This unique feature facilitates the use of native pathways to produce intermediates for desired chemical compounds, akin to metabolic engineering techniques (Figure 1). Moreover, cell-free systems provide a critical advantage over traditional cell-based methods by permitting direct gene programming for necessary enzymes, considerably easing the incorporation of new pathways alongside existing ones [31,32,33]. Integrating designed pathways into cell-free systems can be achieved by combining extracts in which enzymes involved in the pathways have been separately expressed. For example, Kay et al. devised a cell-free metabolic pathway for producing 2,3-butanediol (2,3-BD) by merging extracts enriched with the four enzymes required for the conversion of pyruvate to 2,3-BD (Figure 3). Through careful adjustment of extract volumes, they reported nearly 71% conversion efficiency, nearing the theoretical yield [34]. Similar methodologies were adopted by Grubbe et al. for the production of styrene using a cell-free system derived from *E. coli*. Approximately 40 mM of styrene was produced from phenylalanine by mixing a cell-free synthesis reaction solution expressing phenylalanine ammonia lyase and ferulic acid decarboxylase [35]. Dudley et al. expressed each of the nine different heterologous enzymes required for limonene synthesis using a cell-free synthesis system and then assembled them modularly to rapidly test and optimize them. As a result, the optimal reaction was found to increase limonene yield from 0.2 mM to 4.5 mM [36]. In addition, there are cases of the biosynthesis of various compounds using cell-free synthesis systems, such as mevalonate [37], 1-butanol [38], polyhydroxyalkanoates [39], pinene [40], chlorogenic acid [41], valinomycin [42], and caffeine [43]. These instances underline the cell-free approach’s effectiveness in developing synthetic pathways for optimally producing target compounds. 

**Figure 4:**
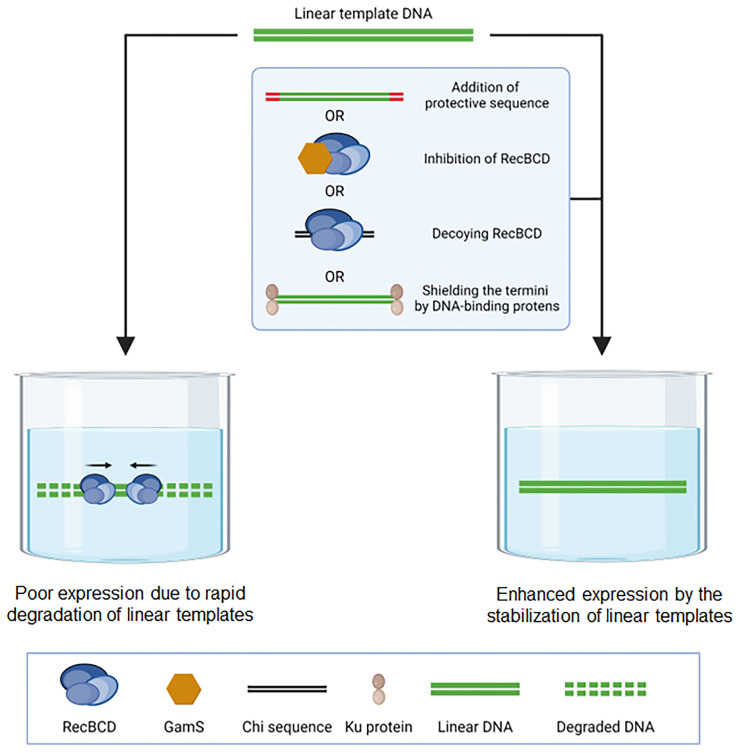
Stabilization of linear DNAs in cell extracts. This figure illustrates the techniques used to stabilize linear DNAs in cell extracts, enhancing their usability in cell-free protein synthesis and thereby increasing the ‘programmability’ of cell-free metabolic engineering systems. While nucleases in cell extracts can rapidly degrade linear DNAs, compromising their stability as templates for protein synthesis, various strategies have been developed to protect linear DNAs from nuclease degradation. These strategies include the use of Gam S, a specific inhibitor of the RecBCD enzyme, the incorporation of chi sequences that decoy the nucleases from the template DNA, and the protection of DNA ends using DNA-binding proteins. Together, these methods significantly improve the durability of linear DNAs in cell-free systems, facilitating their effective use in synthesizing proteins and engineering metabolic pathways.

Achieving a truly ‘programmable’ cell-free metabolic system is made possible by directly expressing the DNA of the required enzymes within the reaction mix. Utilizing PCR-amplified DNA boosts the programmability of cell-free systems, simplifying the Design–Build–Test–Learn (DBTL) cycle by removing the need for time-consuming and labor-intensive cloning and growth phases. Initially, the direct use of PCR products encountered obstacles due to the rapid degradation of linear DNAs by nucleases in the cell extract. However, various methods have been employed to surmount this challenge, including the application of chemical or biological nuclease inhibitors [44], the protection of DNA ends with DNA-binding proteins [45,46], and the use of nuclease substrate sequences as decoys (Figure 4). Thanks to these technical innovations, linear DNAs from PCR or other amplification techniques are now commonly used, achieving translatability on par with plasmid-cloned genes. Leveraging these advancements, Sun et al. successfully demonstrated assembly of various regulatory elements of DNA in the presence of GamS in a cell-free protein synthesis system and rapid prototyping of the assembled genetic circuit [47,48,49].

The capacity for rapid prototyping offered by cell-free synthesis marks a significant advancement in biomanufacturing. It can facilitate the swift development of novel pathways via the DBTL cycle, bypassing the delays inherent in cloning and culturing chassis organisms, and potentially signifies streamlining the development of novel pathways.

## 5. Harnessing Cell-Free Synthesis for Decentralized Biomanufacturing

Cell-free synthesis has matured into a versatile platform that extends beyond recombinant protein production to facilitate the development of innovative metabolic pathways. This method enables the swift evaluation and optimization of pathways, addressing the limitations in speed and throughput inherent in cell-based systems, thereby streamlining the synthesis of chemical products.

The trajectory of cell-free protein synthesis from an analytical instrument to a robust platform for protein production illustrates its potential to transform chemical manufacturing, particularly for applications that require specialized, small-scale production. Traditional chemical manufacturing, which predominantly relies on bulk production in centralized facilities, faces numerous challenges including logistical, financial, operational, and environmental issues. The SARS-CoV-2 global pandemic has underscored the fragility of centralized manufacturing, exposing its susceptibility to supply chain disruptions [50,51]. In biomanufacturing, these vulnerabilities are magnified due to the reliance on a wide range of specialized inputs and the stringent quality control measures essential for biological processes. The scarcity of these inputs, coupled with their critical quality requirements, makes the supply chain exceedingly delicate. Furthermore, the inherent biological characteristics of these processes render them especially vulnerable to environmental variations, adding layers of complexity to their transport and storage.

In contrast, cell-free biomanufacturing presents a strategic alternative, especially in situations where traditional supply chains are compromised by global pandemics, natural disasters, or conflict. By eliminating the need for live cell cultures, cell-free synthesis offers a resilient and flexible manufacturing approach. This technique facilitates decentralized production, significantly reducing the dependence on complex, sensitive biological supply chains and improving accessibility in geographically remote regions or for specialized missions, such as space exploration. Indeed, decentralized cell-free synthesis offers a promising alternative, mitigating these risks by enabling localized production that is adaptable, scalable, and less dependent on complex supply chains. By leveraging cell-free systems, producers can rapidly prototype and produce chemicals and proteins on-site, reducing the dependency on extensive transportation and centralized production facilities. This shift not only enhances the resilience of the manufacturing process but also aligns with sustainable practices by minimizing transportation-related emissions and optimizing resource utilization. As cell-free synthesis continues to evolve, its role in decentralized biomanufacturing could become a cornerstone for more resilient, efficient, and sustainable chemical production industries.

One significant advancement bringing this vision closer to reality is the development of portable cell-free synthesis systems that utilize lyophilized reagents. The necessity of storing reagents at ultra-low temperatures, often below −70 °C, presents a substantial challenge for decentralized biomanufacturing, as it complicates the transport of reagents. However, the dehydration of reaction mixtures offers a solution by enabling the reactivation of the system with just the addition of water on site [52,53]. Although rehydrated cell-free systems have typically shown reduced translational activity compared to their liquid counterparts, innovative methods have been developed to enhance the storability and functionality of lyophilized cell-free systems [54,55]. For instance, Fernando et al. significantly reduced costs by adding lactose as a cryoprotectant and eliminating NTP under the condition of using maltodextrin as an energy source. Katherine et al. synthesized a conjugate vaccine using maltodextrin as an energy source and a cryoprotectant and demonstrated that it could maintain activity for 4 weeks at 50 °C [56,57]. While further research is necessary to enhance the stability of these systems, our results significantly advance the pursuit of storable cell-free systems suitable for decentralized, on-demand protein production.

While ongoing research is crucial to further improve the stability of these systems, these developments mark a substantial stride toward creating storable cell-free systems apt for decentralized, on-demand protein production.

In sum, cell-free biomanufacturing addresses the critical vulnerabilities of traditional biomanufacturing supply chains and signifies a step toward a more sustainable, efficient, and resilient chemical production landscape. As cell-free technology continues to evolve, its adoption in the chemical industry is poised to usher in a new manufacturing era, characterized by environmental stewardship, operational flexibility, and greater global accessibility.

## 6. Conclusions

The urgent call for sustainable development is catalyzing significant transformations across various industrial sectors, primarily focused on reducing carbon dioxide emissions from fossil resource use. Transitioning to renewable energy sources like solar, wind, and tidal power offers viable alternatives for energy generation. However, the chemical production sector requires a unique strategy. Biomanufacturing, which leverages biomass as a raw material, promises to not only address carbon dioxide accumulation but also to enable processes that could be carbon-negative by utilizing emission gases.

To expand the spectrum of chemical products derived from bioprocesses, extensive efforts are required to engineer novel microbial strains with tailored pathways for cost-effective target product synthesis. While optimizing these microorganisms presents challenges, cell-free systems offer a significant alternative by streamlining the expression of essential enzymes for designed processes, thereby circumventing the protracted and labor-intensive genetic engineering and cell cultivation steps typical of cell-based methods.

Advancing cell-free synthesis systems to realize their full potential involves enhancing their programmability and control, particularly for testing designed pathways in a plug-and-play manner. This necessitates a wider range of bio-parts, like synthetic promoters, untranslated regions, and terminators, for precise metabolic flux control. Additionally, creating a diverse repository of cell-free systems from various sources will facilitate the exploration of new pathways and the integration of unique pathways from different strains, advancing synthetic route development.

While challenges persist, employing cell-free systems for pathway development and subsequent integration into host organisms for large-scale production could significantly accelerate biomanufacturing innovation, offering a sustainable avenue for chemical production that aligns with environmental objectives. Furthermore, cell-free synthesis systems might directly produce chemical products, particularly for on-site or customized production needs. With ongoing enhancements in cell-free system yields and efficiency, these possibilities could become realities in the near future.
